# Preventing Viral Contamination: Effects of Wipe and Spray-based Decontamination of Gloves and Gowns

**DOI:** 10.1093/cid/ciz622

**Published:** 2019-09-13

**Authors:** Gwen L Robinson, Stephanie Hitchcock, Zegbeh Kpadeh-Rogers, Nicole Karikari, J Kristie Johnson, Natalia Blanco, Daniel J Morgan, Anthony D Harris, Surbhi Leekha

**Affiliations:** 1 Department of Epidemiology and Public Health, University of Maryland School of Medicine, Baltimore; 2 Department of Pathology, University of Maryland School of Medicine, Baltimore

**Keywords:** personal protective equipment, contamination, decontamination, aerosol

## Abstract

We conducted a laboratory simulation to evaluate the contamination of environmental surfaces when using wipe vs spray methods of personal protective equipment (PPE) decontamination. We did not observe any environmental contamination with the bacteriophage MS-2 when bleach solution spray or wipes were used for PPE disinfection.

## Teaser

In this laboratory simulation to evaluate contamination of nearby environmental surfaces when using wipe vs spray methods of decontaminating personal protective equipment, we did not observe any environmental contamination with bacteriophage MS-2 (Ebola virus surrogate) using bleach spray or wipes.

During the 2014 Ebola outbreak in West Africa, personal protective equipment (PPE) and the environment were frequently decontaminated using disinfectant (usually bleach) spray solutions [1, 2]. In the United States, the Centers for Disease Control and Prevention primarily recommended disinfectant wipes for healthcare worker PPE decontamination [3] due to occupational health concerns of bleach spray exposure such as adverse respiratory effects [4] and potential falls due to slippery conditions created by spraying. An additional concern with spraying contaminated surfaces is potential generation of infectious aerosols [5]. 

In this study, we conducted a laboratory simulation to evaluate the contamination of environmental surfaces when using wipe vs spray methods of PPE decontamination. A secondary objective was to assess reduction in PPE viral contamination with the 2 methods.

## METHODS

**Figure 1.**
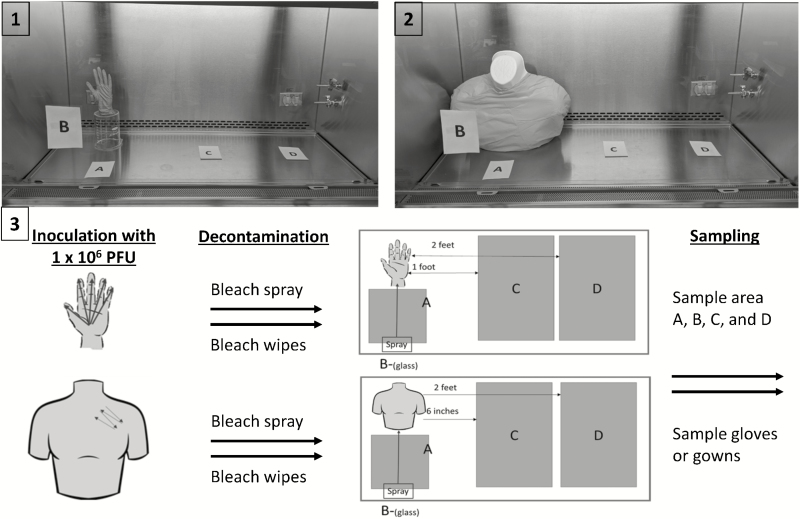
(*1*) Gloved hand manikin under hood. (*2*) Gowned manikin under hood. (*3*) Relationship of manikins with sampling of hood areas A (floor immediately in front), B (front glass), C (floor immediately to right), and D (floor farther to the right). Abbreviation: PFU, plaque-forming unit.

In our study, we used the bacteriophage MS-2, a nonenveloped virus widely used as a conservative surrogate for the enveloped Ebola virus [6]. Experiments were performed using 2 manikins—a hand manikin donning a glove and a chest/torso manikin donning a gown, both placed in a hood (Figure 1). Gloves and gowns were those used for routine patient care in the hospital setting. Separate experiments were performed on the gloved hand and the gowned chest/torso with bleach spray (Clorox Healthcare, Oakland, CA) and Dispatch bleach wipes (Clorox Healthcare, Oakland, CA). A total of 20 experiments were conducted with the following PPE and disinfecting method combinations: glove and bleach spray (n = 5), glove and bleach wipe (n = 5), gown and bleach spray (n = 5), and gown and bleach wipe (n = 5). In each experiment, the manikin PPE (palmar surface of glove or front of gown; Figure 1) was inoculated with 50 µL of the bacteriophage for a final concentration of 1 × 10^6^ plaque-forming units (PFUs). The inoculum was allowed to dry, and samples were collected as described below.

### Positive Glove/Gown Control

The manikin was sampled without intervention. This served as the baseline viral (bacteriophage) load.

### Negative Environmental Control

The surrounding surfaces were sampled in the absence of intervention to ensure no inadvertent contamination of the environment at baseline from the manikin inoculation and set-up process. The following 4 areas of the immediate environment were sampled (Figure 1): (A) a 12 inch × 12 inch area on the floor of the hood immediately in front of the manikin; (B) a 12 inch × 12 inch area on the inside of the glass on the front of the hood; (C) a 12 inch × 24 inch area on the floor of the hood immediately to the manikin’s left; and (D) a 12 inch × 24 inch area on the floor of the hood furthest from and to the manikin’s left.

The manikin PPE was then decontaminated with a bleach wipe or spray by a research team member who make sure the manikin PPE remained wet for the manufacturer-recommended contact time of 1 minute. For wiping, a single bleach wipe was used to decontaminate the palmar surface of the gloved manikin hand or the front of the gowned manikin torso, ensuring coverage of all inoculated surfaces using a continuous wiping action for 1 minute. For spraying, the palmar surface of the gloved manikin hand or front of the gowned manikin torso was sprayed 5 times in succession, aiming to cover all inoculated PPE surfaces and ensuring that a wet surface was maintained for the manufacturer-recommended contact time of 1 minute. Similar to decontamination for Ebola in the field, no surface was wiped dry following spray-based decontamination.

Following the decontamination step, the same environmental surfaces were sampled between 2 to 5 minutes postdecontamination to examine contamination of the immediate environment that may have resulted from bacteriophage particles being dispersed during the decontamination process. The airflow of the hood was switched off prior to the decontamination step to prevent any dispersed bacteriophage from being transported out of the hood by air currents. Environmental surface sampling was conducted as a surrogate for actual air sampling [7, 8]. Finally, manikin PPE was sampled in a standardized manner using a 3M Sponge-Stick with 10 mL neutralizing buffer (St. Paul, MN) in order to analyze bacteriophage load reduction following decontamination.

### Microbiologic Methods

Sponge-sticks were processed using previously described methods, except samples were concentrated by centrifuge at 2700 × *g* for 20 minutes [9]. Quantitative and qualitative cultures were performed for MS-2 following US Environmental Protection Agency protocols [10]. Briefly, for quantitative cultures, serial dilutions of the eluent were made in tryptic soy broth with ampicillin/streptomycin. Each dilution was plated by adding 100 µL of the host bacteria (*Escherichia coli* Famp ATCC 700891) and 500 µL of the dilution to the top agar (0.7% tryptic soy agar with ampicillin/streptomycin). Tubes were gently mixed by swirling and poured onto the 1.5% tryptic soy bottom agar plate. Plates were allowed to dry, inverted, and incubated at 37°C for 16–24 hours. PFUs were counted the following day. For qualitative cultures, 10 µL of the original eluent was plated on a spot plate. Plates were inverted and incubated overnight at 37°C for 16–24 hours. The presence or absence of plaques on the plate was noted the following day.

### Statistical Methods

Differences between median PFUs of MS-2 before and after decontamination (bacteriophage load reduction) were evaluated using the Kruskal-Wallis test.

This study was approved by the University of Maryland Institutional Review Board.

## RESULTS

The median PFU count recovered from gloves and gowns after initial inoculation (positive control) was 5.5 × 10^4^ (interquartile range [IQR], 4.5 × 10^4^ –2.4 × 10^5^). Postdecontamination, MS-2 was not detected on any surrounding environmental surface in quantitative or qualitative cultures in all 20 experiments. Reduction in median MS-2 load from the positive control was noted on gloves and gowns after bleach wipes (4.9 × 10^4^ PFU; IQR, 1.3 × 10^4^–1.1 × 10^5^; *P* = .04) and bleach spray were used (4.7 × 10^4^ PFU; IQR, 3.0 × 10^4^–2.0 × 10^5^; *P* = .01), with no difference in quantitative reduction between wipe vs spray decontamination. A somewhat greater reduction was observed for gloves (1.3 × 10^5^ PFU; IQR, 4.4 × 10^4^–3.6 × 10^5^) vs gowns (9.0 × 10^3^ PFU; IQR, 3.6 × 10^3^–5.2 × 10^4^), but this was not statistically significant.

## DISCUSSION

In this simulation study, we did not observe any environmental contamination of nearby surfaces with bacteriophage MS-2, a human virus surrogate, when bleach solution spray or wipes were used for PPE disinfection. These findings have implications for selection of wipe vs spray-based disinfectants for PPE decontamination, particularly with regards to the concern for aerosolization of infectious pathogens during the spraying process. While we did not directly study aerosol creation, the lack of recovery of the bacteriophage from surrounding surfaces in close proximity (within 1–2 feet) supports a relative lack of significant air and environmental contamination from these decontamination methods, particularly spray-based disinfection.

We also found spray and wipe disinfection of PPE to be equally effective based on the outcome of bacteriophage load reduction. However, it should be noted that despite quantitative reduction, bacteriophage could be recovered from the PPE surfaces following disinfection in nearly all instances. This supports the current recommendations [3] for highly virulent pathogens such as Ebola that call for extremely careful, protocol-based, guided doffing of PPE, where decontamination is used as an adjunct risk-reduction step in the doffing process.

Our use of a hood provided an innovative way of sampling surfaces in the immediate environment to help understand potential airborne spread without conducting more resource-intensive air sampling. Surface sampling has been previously described as an alternative to air sampling to study airborne viruses [8, 11]. Limitations of this study include potentially delayed settling of aerosols that may not have been detected through surface sampling and low-level contamination below the limit of detection of our sampling and culturing methods. Finally, this study was not designed to evaluate potential occupational health hazards of spray disinfection, which should be factored into the development of healthcare cleaning and disinfection protocols.

## Notes

***Financial support..*** This work was supported by the CDC–Prevention Epicenters Program (grant 1U54CK000450-01 to G. L. R., S. H., Z. K. R., N. K., J. K. J., N. B. H., D. J. M., A. D. H., and S. L.).

***Supplement sponsorship.*** This article appears as part of the supplement “Personal Protective Equipment for Preventing Contact Transmission of Pathogens: Innovations from CDC’s Prevention Epicenters Program,” sponsored by the CDC’s Prevention Epicenters Program.

***Potential conflicts of interest.*** J. K. J. reports grants from the University of Maryland, Baltimore, during the conduct of the study. D. J. M. reports grants from the CDC, National Institutes of Health, Agency for Healthcare Research and Quality, and U.S. Department of Veterans Affairs Health Services Research & Development; payments from the Infectious Diseases Society of American and Society for Healthcare Epidemiology of America for expenses to organize or present at national meetings; and honoraria for textbook/journal editing from Springer Nature Inc outside the submitted work. All other authors report no potential conflicts of interest. All authors have submitted the ICMJE Form for Disclosure of Potential Conflicts of Interest. Conflicts that the editors consider relevant to the content of the manuscript have been disclosed.
